# Urbanization and Mental Health in China: Linking the 2010 Population Census with a Cross-Sectional Survey

**DOI:** 10.3390/ijerph120809012

**Published:** 2015-07-31

**Authors:** Juan Chen, Shuo Chen, Pierre F. Landry

**Affiliations:** 1 Department of Applied Social Sciences, The Hong Kong Polytechnic University, Hong Hum, Kowloon, Hong Kong; E-Mail: rockfirm163@gmail.com; 2 Global China Studies, New York University Shanghai, 1555 Century Ave, Pudong, Shanghai 200122, China; E-Mail: pierrelandry@gmail.com

**Keywords:** China, urbanization, mental health, spatial variation, survey, population census

## Abstract

Along with the rapid urbanization in China, the state of mental health also receives growing attention. Empirical measures, however, have not been developed to assess the impact of urbanization on mental health and the dramatic spatial variations. Innovatively linking the 2010 Chinese Population Census with a 2011 national survey of urban residents, we first assess the impact of urbanization on depressive symptoms measured by the Center of Epidemiological Studies Depression Scale (CES-D) of 1288 survey respondents. We then retrieve county-level characteristics from the 2010 Chinese Population Census that match the individual characteristics in the survey, so as to create a profile of the “average person” for each of the 2869 counties or city districts, and predict a county-specific CES-D score. We use this county-specific CES-D score to compute the CES-D score for the urban population at the prefectural level, and to demonstrate the dramatic spatial variations in urbanization and mental health across China: highly populated cities along the eastern coast such as Shenyang and Shanghai show high CES-D scores, as do cities in western China with high population density and a high proportion of educated ethnic minorities.

## 1. Introduction

The state of mental health in China has received growing attention in the past decade due to the increase in the population’s economic and societal stress [1,2]. According to the psychiatric epidemiological surveys conducted by the World Health Organization (WHO) in 2001–2002, the estimated prevalence of mental disorders in the preceding year in two Chinese cities—9.1% in Beijing and 4.3% in Shanghai—was still considerably lower than that of other countries (e.g., 18.4% in France, 26.4% in the United States) [3]. A 2004–2005 psychiatric epidemiological study conducted by Phillips *et al*. in four provinces in China showed the adjusted one-month prevalence of any mental disorder was 17.5%; of mood disorders, 6.1%; and of anxiety disorders, 5.6% [4]. In their recent report on the results of the 2010 Global Burden of Diseases, Injuries, and Risk Factors Study, Yang *et al*. provide clear evidence of the prominence of mental disorders among public health concerns in China: mental and behavioral disorders accounted for 9.5% of all disability-adjusted life-years and 23.6% of all years lived with disability (YLD); seven of the top 20 causes of YLD were mental disorders, with major depressive disorder highest on the list [5].

Over the past three decades, China has definitively shed its agrarian image. More than 50% of its population now live in urban areas and work outside of the agricultural sector [6]. There has been massive rural-to-urban migration and the uncontrolled expansion of Chinese cities has rapidly devoured surrounding rural areas. In 2011, the total urban area was 43,603 square kilometers, almost six times of that in 1981 [7,8]. Of the 440 million people who account for the urban growth since 1979, about half are rural-to-urban migrants and the rest are *in-situ* urbanized rural residents [9,10].

The process of urbanization can cause psychological distress and mental disorders, and exacerbate diseases. Urbanization has some health advantages, such as access to improved health care; it also poses substantial health risks, including ambient air pollution, occupational and traffic hazards, poor diet, and reduced physical activity [5]. Urban living, then, has both positive and negative consequences for individuals’ mental health: cities usually provide superior health-care facilities but environmental pollution and unhealthy lifestyles contribute to deteriorating health status [11,12,13,14,15,16]. The negative effects are experienced directly (e.g., through exposure to polluted air and water) and indirectly (e.g., through perception of risk and attendant chronic stress) by both long-term urban residents and the new urbanites [17,18].

Long-term urban residents are likely to demonstrate negative mental health effects because of their increased exposure to a crowded and polluted environment, as well as other risks associated with urban life [19]. For rural-to-urban migrants and *in-situ* urbanized rural residents, mental health problems may be caused, or aggravated, by the stress of adapting to an unfamiliar society or a different lifestyle. Studies on the effects of migration on individuals’ health consistently show that the “healthy migrant” phenomenon does not apply to mental health; instead, the mental health status of migrants is the same or poorer than that of urbanites [20,21,22]. There are few empirical measures of the impact of urbanization on individuals’ mental health. Such measures are particularly crucial in China, where the process of urbanization is still rapidly ongoing and the issue of mental health receives growing attention [8,23].

Linking the 2010 Chinese Population Census with a 2011 national survey of urban residents, we first assess the impact of urbanization on depressive symptoms measured by the Center of Epidemiological Studies Depression Scale (CES-D) among 1288 survey respondents. We then retrieve county-level characteristics from the 2010 Census that match the individual characteristics from the survey, so as to create a profile of the “average person” for each of the 2869 counties or city districts, and to predict a county-specific CES-D score. We use this county-specific CES-D score to further compute the CES-D score for the urban population at the prefectural level, and to demonstrate the dramatic spatial variations in urbanization and mental health across China.

## 2. Methods

### 2.1. Study Design and Data Sources

The individual mental health and socio-demographic data for this study come from the 2011 Migration and Quality of Life Survey we completed in collaboration with the Research Center for Contemporary China (RCCC) at Peking University in May and June of 2011.

We employed spatial probability sampling specifically designed to reach urban residents regardless of their household registration (*hukou*) status [24]. The actual sampling procedure was carried out in several stages. We first randomly selected 26 primary sampling units (PSUs), which are cells of spatial grids defined as half square degrees (HSDs) of latitude and longitude, within strata from a spatial sampling frame of China taken by our partners at RCCC. The strata cover seven geographical areas. In each PSU, we then randomly selected two secondary sampling units (SSUs), which are half square minutes (HSMs) of latitude and longitude, in areas deemed “urban” (We adapted the 2009 Operational Linescan System nighttime light data provided by the Defense Meteorological Satellite Program to the survey sample design and the details are described in another article) [23,25].

From these 26 PSUs and 52 SSUs, spread over 19 provinces, 27 prefectures, and 31 counties or city districts, we randomly sampled 1906 households and successfully interviewed 1288 individuals between the ages of 18 and 70 for a response rate of 67.6%. All interviews were conducted in Chinese, face-to-face by trained local college students as interviewers. The average length of the interviews was 38.3 min. To ensure quality control, our fieldwork supervisors checked each completed questionnaire on site during the stage of data collection. Any abnormal patterns or problems were immediately followed up. After completing the fieldwork and data input, the research staff at RCCC did another round of thorough checking and excluded two questionnaires because the respondents were not properly selected within the household. Survey weights were developed to adjust for unequal probabilities of selection and non-response rates. Post-stratification weights were calculated based on the age and gender distribution of the urban population reported in the 2010 Chinese Population Census [10]. Approval for the ethical review of a research project involving human subjects was granted to Juan Chen by The Hong Kong Polytechnic University.

The short form of the Center of Epidemiological Studies Depression Scale (CES-D), an eight-item questionnaire that measures depressive symptoms experienced during the previous week, was administered in the survey. The CES-D was introduced in China in the 1990s and its validity has been tested in various studies [17,23,26,27]. The final score (the sum of the scores for each response) ranges from 0 to 24, with higher scores indicating higher levels of depressive distress. The Cronbach’s α is 0.75 for the study sample.

The individual demographic information collected in the survey includes age, gender, marital status, and ethnicity. Measures of socio-economic status are education, occupation, and home ownership. Two variables are dichotomously coded for *hukou* status: urban *hukou* and non-local *hukou* (*i.e.*, *hukou* that is not from the county of residence).

We use data from the 2010 Census aggregated at the county level to determine the urbanization measure. Population density is measured by the average number of people per square kilometer in each county or city district. The natural logarithm transformation of population density is used in the subsequent regression analysis.

We retrieve county-level characteristics from the 2010 Census that match individual characteristics identified in the survey, including age, gender, marital status, ethnicity, education, occupation, home ownership, urban *hukou*, and non-local *hukou*, to create the profile of an average person for each of the 2869 counties or city districts. These variables are used to predict county-specific CES-D scores.

### 2.2. Statistical Analysis

We first compute the descriptive statistics of individual characteristics based on the 2011 Migration and Quality of Life Survey and the county-level urbanization measure of the 31 counties or city districts retrieved from the 2010 Census. Twenty cases from the survey are excluded due to missing data, leaving a sample of 1268 for the analysis. We apply survey and post-stratification weights, and address the problems inherent in a multi-layered clustered sampling design by using the “svy” (survey) commands in Stata 12.0, which estimates appropriately corrected standard errors in the presence of stratification and clustering for individual characteristics. We then estimate ordinary least square (OLS) regressions for respondents’ CES-D scores. Individual demographic characteristics, socio-economic status, *hukou* status, and the logarithmic form of the county-level population density are included as the independent variables. Because the CES-D scores are skewed toward lower values, we also run the regressions with a natural logarithm transformation to approximate a normal distribution. Similar findings are observed when CES-D is transformed in natural logarithm.

We further compute the descriptive statistics on selected variables of China’s 2869 county-level administrative units from the 2010 Census, which we use to predict county-specific CES-D scores. Based on the results of OLS regressions with survey data, we plug in the county-level characteristics in the specified equation to calculate the county-specific CES-D score. Because the 2011 Migration and Quality of Life Survey was conducted with a sample of urban residents, we weight each county-specific CES-D score according to the county’s share of the prefectural urban population, compute the predicted CES-D scores for the urban population in each of the 339 prefectures in China, and create a map of predicted prefectural CES-D scores for the urban population.

## 3. Results

In Table 1, we summarize the descriptive statistics of individual CES-D scores and socio-demographic characteristics from the survey and the county-level urbanization measures based on the 2010 Chinese Population Census. The mean CES-D score among the survey respondents is 6.10, with a standard error of 0.56. The average population density of the 31 counties or city districts sampled in the survey ranges from 60.74 to 4168.95 people per square kilometer, with 746.56 as the average.

**Table 1:** Descriptive statistics of individual characteristics and county-level urbanization measure.

	Mean /Percentage	Standard Error	Min	Max
**Individual characteristics (*n* = 1268)**				
CES-D score (0–24, mean)	6.10	0.56	0	24
Age 20–29 (%)	28.10		0	1
Age 30–39 (%)	22.65		0	1
Age 40–49 (%)	26.02		0	1
Age 50–59 (%)	15.41		0	1
Age 60–69 (%)	7.82		0	1
Gender (female, %)	50.06		0	1
Ethnicity (ethnic minority, %)	2.99		0	1
Marital status (married, %)	80.81		0	1
Education (years, mean)	9.86	1.04	0	22
Occupation (professional/managerial, %)	17.48		0	1
Homeowner (%)	78.56		0	1
Urban *hukou* (%)	56.59		0	1
Non-local *hukou* (%)	25.34		0	1
**County-level urbanization measure (*n* = 31)**				
Population density (per square kilometer, mean)	746.56	778.26	60.74	4168.95
Population density (natural logarithm, mean)	6.21	0.96	4.11	8.34

Note: Survey design effects (strata, clusters, and sampling weights) are adjusted in the mean/percentage estimations of individual characteristics.

**Figure 1:**
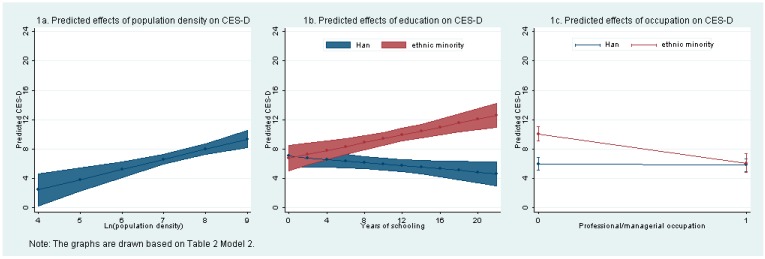
Estimated effects of county population density and individual education and occupation on CES-D scores.

In Table 2, we display results from the two OLS regressions on the CES-D scores with the individual socio-demographic characteristics from the survey and the county-level population density from the 2010 Census as independent variables. The natural logarithm of county-level population density is a consistent, strong, and significant predictor of individual CES-D scores, with coefficient = 1.38 and *p* < 0.001 in both models, which indicates that a one percent increase in county-level population density would lead to 1.38 increase in the mean CES-D score. Figure 1a further demonstrates that as the population density changes from 60.74 to 4168.95 people per square kilometer across the 31 counties or city districts, the average predicted individual CES-D score increases from 2.61 to 8.44, an almost 6-point difference on the CES-D scale of 0–24.

**Table 2:** Ordinary least square (OLS) regression estimation of individual Center of Epidemiological Studies Depression Scale (CES-D) scores (*n* = 1,268).

	Model 1	Model 2
**Individual characteristics**		
Age 20–29 (reference group)	—	—
	—	—
Age 30–39	0.402	0.402
	(0.718)	(0.700)
Age 40–49	1.571	1.631 *
	(0.782)	(0.768)
Age 50–59	0.225	0.270
	(1.051)	(1.043)
Age 60–69	0.242	0.374
	(0.979)	(0.863)
Gender (female)	0.395	0.450
	(0.208)	(0.216)
Marital status (married)	0.099	0.010
	(0.245)	(0.230)
Ethnicity (ethnic minority)	4.515 ***	0.391
	(0.795)	(0.730)
Education (years)	−0.095	−0.108
	(0.057)	(0.060)
Education (years) x Ethnicity (ethnic minority)		0.374 ***
		(0.079)
Occupation (professional/managerial)	−0.311	−0.195
	(0.276)	(0.339)
Occupation (professional/managerial) x Ethnicity (ethnic minority)		−3.778 ***
		(0.795)
Homeowner	0.783	0.835
	(0.603)	(0.619)
Urban *hukou*	−1.063	−1.111
	(0.636)	(0.664)
Non-local *hukou*	−0.621	−0.508
	(0.651)	(0.606)
**County-level urbanization measure**		
Population density (natural logarithm)	1.381 ***	1.379 ***
	(0.314)	(0.321)
**Constant**	−2.904	−2.815
	(2.419)	(2.481)
**Wald F statistics**	14.34 (13,19)	49.78 (15,19)

Notes: Survey design effects (strata, clusters, and individual weights) are adjusted in the model estimations. Coefficients are reported; standard errors in parentheses; * *p* < 0.05, ** *p* < 0.01, *** *p* < 0.001.

Of all the individual socio-demographic characteristics, belonging to an ethnic minority appears to be most strongly associated with high CES-D scores, as shown in Table 2, Model 1 (coefficient = 4.52, *p* < 0.001). To further investigate the actual association between belonging to an ethnic minority and CES-D score and to improve the model specification, in Model 2, we allow two variables to interact with ethnic minority: years of education and professional/managerial occupation. The coefficient on ethnic minority is no longer significant in Model 2, whereas both coefficients on the interaction terms are strong and significant. The positive coefficient on the interaction between ethnic minority and education (coefficient = 0.37, *p* < 0.001) indicates that those belonging to ethnic minorities with more years of schooling are likely to have higher CES-D scores, whereas the negative coefficient on the interaction between ethnic minority and occupation (coefficient = −3.78, *p* < 0.001) signifies that holding a professional or managerial occupation significantly reduces the CES-D scores of ethnic minorities. The result from the adjusted Wald F test indicates that including the two interaction terms in Model 2 creates a statistically significant improvement in the fit of the model. In Figure 1b and Figure 1c, we show the differential marginal effects of education and occupation on CES-D scores for respondents belonging to ethnic minorities and Han respondents. 

**Figure A1:**
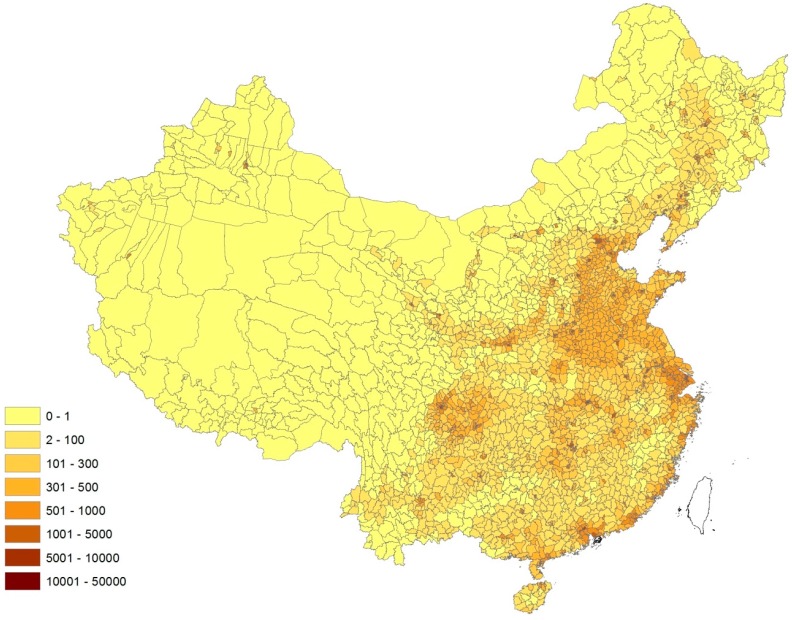
Map of county population density in China (*n* = 2869).

We now move on to predicting county-specific CES-D scores. In Table 3, we first present the descriptive statistics on selected county variables from the 2010 Chinese Population Census. These county-level variables are calculated as shares of the relevant individual socio-demographic attributes shown in Table 1. The descriptive results demonstrate huge variations among the 2869 county-level administrative units in China. The measure of urbanization (*i.e.*, the population density), in particular, ranges from 0.12 to 47,181.50 people per square kilometer, averaging 1258.34 people per square kilometer (see Figure A1). The percentage of the residents belonging to ethnic minorities ranges from 0 to 99.78, with an average of 16.23.

**Table 3:** Descriptive statistics of county characteristics, county-level urbanization measure, and predicted county and prefectural CES-D scores.

	Mean	Standard Deviation	Min	Max
**County characteristics (*n* = 2869)**				
Age 20–29 (%)	23.50	5.88	8.39	51.31
Age 30–39 (%)	23.50	3.16	10.42	42.03
Age 40–49 (%)	25.22	3.06	14.46	64.71
Age 50–59 (%)	17.11	3.42	3.92	27.41
Age 60–69 (%)	10.67	2.44	0.35	20.12
Gender (female, %)	48.69	1.44	28.39	57.88
Ethnicity (ethnic minority, %)	16.23	29.00	0.00	99.78
Marital status (married, %)	71.40	5.46	37.42	82.31
Education (years, mean)	8.71	1.47	2.00	13.14
Occupation (professional/managerial, %)	5.46	3.35	0.00	22.60
Homeowner (%)	87.60	11.98	1.23	100.00
Urban *hukou* (%)	29.53	23.56	1.58	99.40
Non-local *hukou* (%)	5.47	11.42	0.00	88.69
**County-level urbanization measure (*n* = 2869)**				
Population density (per square kilometer, mean)	1258.34	3717.72	0.12	47,181.50
Population density (natural logarithm, mean)	5.56	1.86	−2.15	10.76
**Predicted county CES-D score (*n* = 2869)**	5.57	2.03	−6.50	11.46
**Predicted prefectural CES-D score (*n* = 339)**	5.85	1.67	−1.80	9.24

We next use the coefficients obtained from the OLS regression reported in Table 2, Model 2, to predict county-specific CES-D scores, drawing on county-level data from the 2010 Census. Here we treat each county-level unit as a county average person, which means for example the percentage of ethnic minorities of any county-level unit ranging from 0% to 99.78% is regarded as the probability of a county average person being an ethnic minority ranging from 0 to 0.9978. Based on the OLS results reported in Table 2, Model 2, we plug the indicators for each county average person into the following equation to obtain the predicted county-specific CES-D score for each of the 2869 county-level units. The resultant county CES-D scores, as shown in the second to the last row of Table 3, range from −6.50 to 11.46, with an average of 5.57 and a standard deviation of 2.03.

$$\hat{y}=\alpha +\;\sum^{\text{​}}\beta_{i}x_{i}$$

$\hat{y}$ is the predicted county-specific CES-D score;
*α* is the constant of the OLS regression reported in Table 2, Model 2;
*β_i_* is the vector of coefficients obtained from Table 2, Model 2; and
*x_i_* is the vector of values on county characteristics presented in Table 3.

The indicators of county characteristics that match the individual socio-demographic attributes in the survey all have values within the expected data range. The population densities of the 2869 county-level units whose natural logarithm ranges from −2.15 to 10.76 are out of the bounds of the 31 counties or city districts in the survey ((4.11, 8.34) in natural logarithm). To be more specific, there are 433 counties with population density in natural logarithm lower than 4.11 and 177 counties with population density in natural logarithm higher than 8.34. Because of the prediction made out of sample, we observe negative values on the predicted CES-D scores for 53 counties with particularly low population density. The predicted county-specific CES-D scores for places with high population density are reasonable and within the expected range. To further measure the uncertainty of the predicted county-specific CES-D scores, we calculate the standard error of the prediction, which ranges from 0.24 to 3.41, with an average of 0.78 and a standard deviation of 0.42. As expected, counties with lower population densities tend to have higher standard errors for the predicted county specific CES-D scores. Particularly, the average standard error of the prediction for the 433 counties with population density in natural logarithm lower than 4.11 is 1.57, whereas the average standard error of the prediction for the 177 counties with population density in natural logarithm higher than 8.34 is 0.63, almost the same as the average standard error of the prediction for the 2259 counties with population density in natural logarithm within the bounds of (4.11, 8.34) which is 0.64.

**Figure 2:**
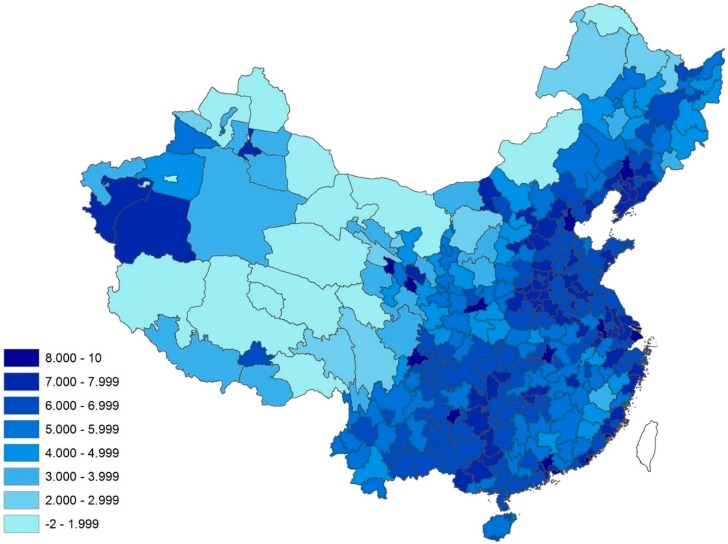
Map of predicted prefectural CES-D scores for the urban population (*n* = 339).

Since the 2011 Migration and Quality of Life Survey only sampled urban residents, we further weight each predicted county-specific CES-D score according to the county’s share of the prefectural urban population, and compute the predicted CES-D scores of the urban population for each of the 339 prefectural administrative units. The resultant prefectural CES-D scores, reported in the last row of Table 3, range from −1.80 to 9.24, with an average of 5.85 and a standard deviation of 1.67. Fourteen cities, including Shenyang, Shanghai, Guiyang, and Xining, have predicted CES-D scores greater than 8. In Figure 2, we map out the predicted prefectural CES-D scores. The figure clearly shows a few clusters with high CES-D scores: metropolises such as Shenyang and Shanghai along the eastern coast with high population densities have high scores, as are cities in western China with high population densities and a high proportion of educated ethnic minorities such as Guiyang and Xining.

## 4. Discussion and Conclusions

There is no doubt that China will continue to urbanize rapidly [28]. With the urbanization of the countryside outstripping the urbanization of the people, there is an urgent need to determine the mental health effects of this phenomenon. Such measures are particularly crucial in China where the level of urbanization is expected to further increase and mental health problems are also on the rise. Using population density as a measure of urbanization, we estimate the effect of urbanization on residents’ mental health: the county-level population density appears to be a consistent, strong, and significant predictor of individual CES-D scores.

Innovatively linking the 2010 Chinese Population Census with the survey data, we further estimate the CES-D scores for each of the 2869 county “average person” and compute the prefectural CES-D scores for the urban population. The resultant map in Figure 2 demonstrates clearly the dramatic spatial variations in urbanization and mental health across China. Large metropolitan cities along the eastern coast such as Shenyang and Shanghai have a much greater likelihood of high CES-D scores. Because the population densities of the 2869 county-level units are out of the bounds of the 31 counties or city districts in the survey, we observe some negative values on the predicted county-specific CES-D scores. Such issue could only be addressed when survey data on mental health are available from more counties, particularly those of low population density. The predicted county-specific and prefectural CES-D scores for places with high population density, nonetheless, fall within the expected data range and are robust according to the uncertainty test.

The potential effects of the unprecedented urbanization of the world’s most populous nation on mental health require further study and policy attention. Properly designed and managed urbanization can lead to improvements in environment and health, but rapid, unplanned, and unregulated urbanization creates environmental pollution and health problems [29]. The results of this study suggest that measures to promote mental health and prevent mental disorders among the urban population should first target cities with high population densities in both eastern and western China. In the meantime, the Chinese government investment promoting urbanization has concentrated on large cities, where the urban population keeps growing and the issue of mental health becomes increasingly prominent. To relieve the population and mental health pressures exerted on large cities, the government must achieve a more equal distribution of resources and opportunities between large and small cities so as to improve the living conditions and opportunities of residents in small cities and reduce the flow of population migration to large cities.

The findings of our study also draw attention to high CES-D scores in cities with a high proportion of educated ethnic minorities in western China. There is hardly any research on the mental health status of ethnic minority groups in China [30]. We speculate that the high CES-D scores may be caused by the barriers that ethnic minorities face in obtaining professional/managerial jobs, even after higher education. This argument, however, needs to be verified by further empirical investigation.

## References

[1] Park L., Xiao Z., Worth J., Park J.M. (2005). Mental health care in China: Recent changes and future challenges. Harv. Heal. Policy Rev..

[2] Shen Y., Zhang M., Huang Y. (2006). Twelve-month prevalence, severity, and unmet need for treatment of mental disorders in metropolitan China. Psychol. Med..

[3] World Health Organization (WHO) (2004). World Mental Health Survey Consortium. Prevalence, severity, and unmet need for treatment of mental disorders in the world health organization world mental health surveys. J. Am. Med. Assoc..

[4] Phillips M.R., Zhang J., Shi Q., Song Z., Ding Z., Pang S. (2009). Prevalence, treatment, and associated disability of mental disorders in four provinces in China during 2001–05: An epidemiological survey. Lancet.

[5] Yang G., Wang Y., Zeng Y., Gao G.F., Liang X., Zhou M. (2013). Rapid health transition in China, 1990–2010: Findings from the Global Burden of Disease Study 2010. Lancet.

[6] National Bureau of Statistics of China (2012). China Statistical Yearbook 2012.

[7] Ministry of Housing and Urban-Rural Development of China (2012). China’s Urban Construction Statistical Yearbook 2011.

[8] Yeh A.G.O., Xu J., Liu K. (2011). China’s Post-Reform Urbanization: Retrospect, Policies and Trends.

[9] Chan K.W., Ness I., Bellwood P. (2013). China, Internal Migration. The Encyclopedia of Global Human Migration.

[10] National Bureau of Statistics of China (2012). Tabulation on the 2010 Population Census of the People’s Republic of China.

[11] Galea S., Ahern J., Rudenstine S., Zachary W., Vlahov D. (2005). Urban built environment and depression: A multilevel analysis. J. Epidemiol. Community Health.

[12] Li X., Wang C., Zhang G., Xiao L., Dixon J. (2012). Urbanisation and human health in China: Spatial features and a systemic perspective. Environ. Sci. Pollut. Res..

[13] Macintyre S., Ellaway A., Cummins S. (2002). Place effects on health: How can we conceptualise, operationalise and measure them?. Soc. Sci. Med..

[14] Moore M., Gould P., Keary B.S. (2003). Global urbanization and impact on health. Int. J. Hyg. Environ. Health.

[15] World Health Organization (WHO) (2008). Our Cities, Our Health, Our Future: Acting on Social Determinants for Health Equity in Urban Settings.

[16] World Health Organization (WHO), United Nations Human Settlements Programme (UN Habitat) (2010). Hidden Cities: Unmasking and Overcoming Health Inequities in Urban Settings.

[17] Chen J., Chen S., Landry P.F. (2013). Migration, environmental hazards, and health outcomes in China. Soc. Sci. Med..

[18] Peek M.K., Cutchin M.P., Freeman D., Stowe R.P., Goodwin J.S. (2009). Environmental hazards and stress: Evidence from the Texas City Stress and Health Study. J. Epidemiol. Community Health.

[19] Gong P., Liang S., Carlton E.J., Jiang Q., Wu J., Wang L. (2012). Urbanisation and health in China. Lancet.

[20] Chen J. (2011). Internal migration and health: Re-examining the healthy migrant phenomenon in China. Soc. Sci. Med..

[21] Li L., Wang H., Ye X., Jiang M., Lou Q., Hesketh T. (2007). The mental health status of Chinese rural-urban migrant workers. Soc. Psychiatry Psychiatr. Epidemiol..

[22] Li X., Stanton B., Fang X., Xiong Q., Yu S., Lin D. (2009). Mental health symptoms among rural-to-urban migrants in China: A comparison with their urban and rural counterparts. World Health Popul..

[23] Chen J., Chen S., Landry P.F., Davis D.S. (2014). How dynamics of urbanization affect physical and mental health in urban China. China Q..

[24] Landry P.F., Shen M. (2005). Reaching migrants in survey research: The use of the global positioning system to reduce coverage bias in China. Polit. Aanl..

[25] National Bureau of Statistics of China National standard definition of urban and rural. http://www.stats.gov.cn/zjtj/tjbz/tjyqhdmhcxhfdm/2011.

[26] Boey K.W. (1999). Cross-validation of a short form of the CES-D in Chinese elderly. Int. J. Geriatr. Psychiatr..

[27] Zhang B., Fokkema M., Cuijpers P., Li J., Smits N., Beekman A. (2011). Measurement invariance of the Center for Epidemiological Studies Depression Scale (CES-D) among Chinese and Dutch elderly. BMC Med. Res. Method..

[28] State Council of China Suggestions for advancing the reform of the Household Registration System. State Council [2014] No. 25 2014. http://www.chinanews.com/gn/2014/07-30/6439778.shtml.

[29] Zhu Y.G., Jones K.C. (2010). Urbanisation and health in China. Lancet.

[30] Xiang Y.T., Xu X., Sartorius N., Ungvari G.S., Chiu H.F.K. (2012). Mental health in China: Challenges and progress. Lancet.

